# Lifestyle and Long-Term Smoking Cessation

**DOI:** 10.1177/1179173X20963062

**Published:** 2020-10-13

**Authors:** Tove Sohlberg, Karin Helmersson Bergmark

**Affiliations:** Department of Sociology, Stockholm University, Stockholm, Sweden

**Keywords:** Lifestyle, smoking cessation, long-term smoke free, Swedish health care

## Abstract

**Background:**

Since smoking is the leading cause of preventable death, discouraging smoking initiation, encouraging smoking cessation, and exploring factors that help individuals to stay smoke free are immensely important. One such relevant factor may be the impact of lifestyle for long-term smoking cessation.

**Method:**

A representative sample of successful quitters was recruited for a study about smoking cessation. These respondents are now part of a 7-year follow-up with the overall aim of revealing factors affecting long-term smoking cessation. Descriptive analyses were carried out at baseline and at follow-up, as well as a further two-step cluster analysis to explore profiles of long-term smoke-free individuals.

**Results:**

A majority did not make any particular lifestyle changes, but among those who did, most adopted a healthier lifestyle and/or increased their quota of physical training, where permanent changes in this direction seem to promote a more enduring smoke-free life.

**Conclusions:**

Individuals who want to quit smoking should be encouraged to increase their level of physical activity. Swedish health care institutions should be able to provide support for this both initially and over time to promote the long-term maintenance of a smoke-free lifestyle.

## Introduction

The lifestyle that we are reared in or that we embrace when independently forming our lives is a powerful factor that should always be included in discussions and analyses of long-term personal development. Smoking constituted during the last century an important symbolic gadget, suggesting the user’s free and modern lifestyle. As was proven, smoking came with a high price for our health. Even though smoking as lifestyle today may be somewhat outdated, smoking is still a global public health problem, annually killing about 8 million individuals,^1^ whereof about 12 000 Swedes.^2^ Naturally, discouraging smoking initiation, encouraging smoking cessation and exploring factors that help individuals to stay smoke free is of great interest for the society at large. Most studies in this field of research focus on long-term effects of medical or behavioral treatment of smokers. In this study, however, we are interested in lifestyle habits that may facilitate or obstruct long-term smoking cessation.

### Preparing for smoking cessation

Much of the existing research focus on the effects of different strategies for smoking cessation, such as using the medicinal drug Bupropion SR,^3^ Nicotine Replacement Therapies (NRTs),^4^ e-cigarettes,^5^ and the Swedish moisturized tobacco known as snus.^6^ There are also comparisons regarding the effectiveness of these strategies, such as between snus and NRTs,^7^ between e-cigarettes and NRTs,^8^ and the use of NRTs by gender.^9^

Beyond medications and nicotine replacements there is a plethora of behavioral tools such as psychotherapy, hypnosis and face-to-face interventions via health care or quit-smoking groups. Telephone counseling (eg, Quit-lines) has shown to be an important support system, and even more so if callback counseling is included.^10^ Previous studies have also focused on the impact of certain life events such as pregnancy^11^ or major surgery, for successful cessation to take place.^12^ A standard procedure before surgery nowadays is to demand that the patient quits smoking. Such so-called teachable moments can both serve as immediate motivation and lead to improved health overall. Moreover, Sweden as a welfare state has a well-developed health care system that besides handling tobacco-related ill-health also provides readily available advice on how to become smoke free. The next step is then to stay smoke-free but there is a lack of advice on how to keep it up over time.

### Lifestyle habits and smoke-free maintenance

Previous studies on lifestyle habits and smoking cessation often link to a medical condition, for example, diabetes^13^ or cardiovascular diseases^14^ while others have focused on one factor at a time as shown below. Lifestyle habits are, however, multicomponent behavior including among other things diet, exercise, sleep and alcohol and/or other substance use.^15^

Common reasons to smoke include for example, effects such as mood enhancement, anger reduction, and stress management,^16^ and cessation may produce withdrawal symptoms, such as anxiety, irritability, restlessness, insomnia, increased hunger, and increased eating.^17^ Many women smokers argue that they smoke in order to control their weight.^15,17^

It has been shown that exercise has a general positive influence on anxiety and stress (especially lifestyle-related stress) along with mood state.^18^ A Cochran review of exercise-based interventions for abandoning smoking habits found no evidence of exercise aiding long-term smoking cessation, but this may be due to bias in trials and publications.^19^ Other studies have found moderate exercise to enhance short-term smoking cessation among women,^20^ but also improvement of long-term smoke-free maintenance.^21,22^

Women tend to gain more weight than men do when they give up smoking,^23^ reasonably related to the follow-on effects of increased hunger and increased eating, so many women resume smoking in the interests of weight control.^15,16^ Further, alcohol consumption and tobacco use are closely linked behaviors.^24^ Especially hazardous drinking patterns may be a risk factor for smoking and relapse,^25^ but even low to moderate alcohol consumption increases the risk of smoking^26^ and may decrease the chances of successful smoking cessation.^27^ Hence, a change in drinking habits could be thought to facilitate a more long-term smoke-free life.

By taking several different components of lifestyle habits into account this study adds to the existing knowledge on what works when quitting, and more importantly, what works for maintaining a long-term smoke-free life.

### Aims

The general aim of the present study is to examine the association between reported changes in lifestyle factors and continued smoking cessation among originally 1 year successful quitters. Therefore, we will be examining in more detail whether changes in health-related behavior as a means to smoking cessation – including physical training and eating habits – is associated with whether or not people come to maintain a smoke/nicotine-free lifestyle.

## Material and Method

### Data

**Figure 1.**
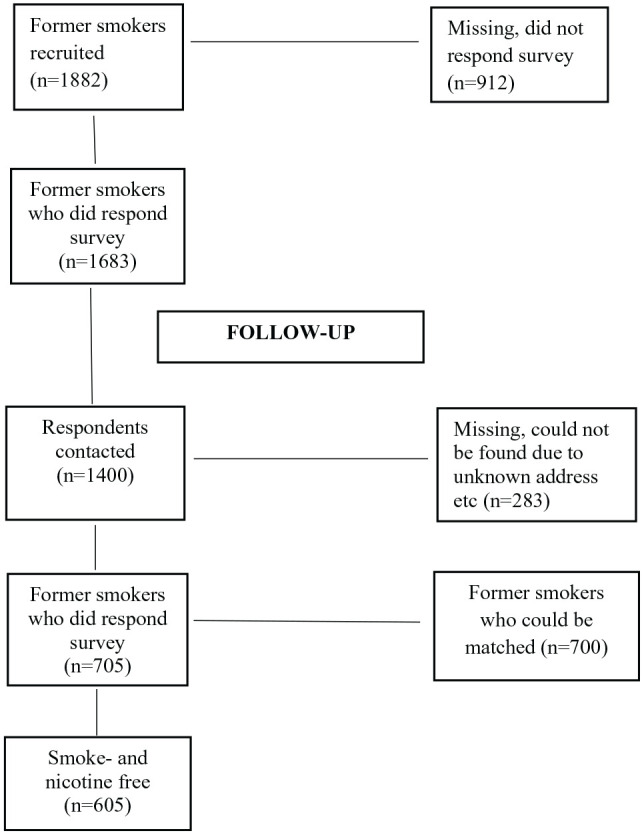
Flow chart showing respondent sample size.

The respondents were originally recruited from the 2000 to 2012 Monitor Project, a running monthly survey directed to a representative sample of 1500 respondents aged 16 to 84 (18 000 per year) and conducted at the Centre for Social Research on Alcohol and Drugs (SoRAD, now within the Department of Public Health Sciences at Stockholm University). During the period October 2009 to May 2010 a sample of former smokers (n = 1882) was recruited for a study about smoking cessation. The response rate was over 89% (n = 1683) and these respondents are now part of this 7-year follow-up with the overall aim of revealing factors affecting long-term smoking cessation on an individual level (see Figure1). Both studies were approved by the Regional Ethical Review Board in Stockholm (2009/2102-31/5; 2017/561-31/5) and the respondents provided informed consent in the web-survey.

The initial sample was recruited from among the general population and a previous study has shown no difference in tobacco use between non-respondents and a concurrent sample of respondents.^28^ For various reasons (unknown address, moved abroad, deceased) 283 individuals could not be found for the second data collection 7 years later, so in total 1400 respondents were contacted regarding participation and asked to answer a web-based survey that ran from August 2017 to February 2018. The response rate was about 50% (n = 705), but a non-response analysis on the follow-up showed no significant differences between participants and non-participants in such sample characteristics as gender, age, education or economic status.

In the present study 700 follow-up respondents were matched with themselves at baseline. Five of these had stated the wrong serial number (given at T1) and therefore could not be matched. A vast majority (86%) was still smoke free and nicotine free. This majority of resilient non-smokers is in itself an interesting result and might surprise, but among former smokers there seems to be a non-use stability that is more long-term than might have been expected, even though relapses can be expected to be more prevalent in the dropout group.

### Variables

#### Background

Most questions had pre-coded alternatives for the respondents to choose from. The background characteristics at baseline are *sex, education, age when quitting smoking* and *present age*, where education was measured as highest degree achieved. Age when quitting smoking is self-reported by the respondents in retrospect.

#### Lifestyle factors

*Drinking habits* were measured at both first and second examination, partly by frequency of drinking alcohol: from never, once a month, once a week, several times a week to daily, and partly by the frequency of so-called binge-drinking. *Binge drinking* was measured with the question “How often. . . drink as much alcohol as equal to at least a bottle of wine (75 cl) or 5 shots (25 cl) or 4 cans of beer at one and the same time?” with the alternatives weekly, sometime every month, a few times a year or never.

*Lifestyle habits* were measured with a given set of alternatives (see Appendix for questions and answer options at baseline and follow-up). In the baseline survey the respondents were asked to what extent these had contributed to their success with the smoking cessation. The alternatives covered a healthy diet, good sleeping habits and meaningful leisure time (healthy lifestyle), as well as starting up or intensifying physical training (exercise). The respondents were also asked whether they had increased their consumption of food, pastries, candy, desserts (eating). There were such options as well as avoiding situations where smoking was permitted, changing drinking habits and altered social relations. All answers were then categorized into Yes (to a great extent/to some extent) and No (quite small extent/not at all).

In the follow-up, the respondents who had stated at baseline that they had made any or several of these lifestyle changes were asked whether any of these factors had become a permanent lifestyle adjustment (yes/no).

### Analyses

First, in order to give a description of the respondents’ background at baseline, as well as their current age, a percentage distribution is shown in Table 1. Frequency of drinking habits and binge drinking at baseline respectively at the follow-up is shown in Table 2. Moreover, a descriptive analysis of reported changes at baseline and implemented changes at follow-up is shown in Table 3.

**Table 1.** Background characteristics at first examination (baseline) (%).

	Baseline (n = 700)
Sex	
Women	54.4
Men	45.6
Education	
Primary school	16.8
Secondary school	41.4
University	41.7
Age when quitting smoking (mean)	40 (std 12)
Present age (mean)	66 (std 11)

* std = standard deviation.

**Table 2.** Drinking habits at baseline and at follow-up (%).

Change in drinking habits (yes)	Baseline	Follow-up
Alcohol use	(n = 591)	(n = 581)
Never	7.8	9.3
Once a month	20.5	17.9
Once a week	33.7	31.5
2-6 times a week	37.1	38.6
Daily	1.0	2.8
Binge	(n = 592)	(n = 591)
Never	36.1	55.2
Weekly	9.5	8.0
Sometimes every month	18.6	8.3
A few times per year	35.8	28.6

* alcohol use, chi^2^ 0.074; binge, chi^2^ 0.349.

**Table 3.** Changes in life-style at smoking cessation (baseline) becoming a permanent part of life (follow-up).

	Baseline	Follow-up
Changes in life-style factors	% yes	% yes
General healthier lifestyle	37.4 (n = 220)	30.9 (n = 159)
More physical training	27.9 (n = 163)	18.1 (n = 92)
Eat more (food, sweets)	17.9 (n = 104)	9.6 (n = 48)

Second, all follow-up respondents were included in a two-step cluster analysis to explore profiles (clusters) of long-term smoke-free individuals. This procedure creates typical groups of successful quitters, giving insight in whether a health-related behavior is part of this success or not.

Cases represent objects to be clustered, and the variables represent attributes upon which the clustering is based. This procedure works also with categorical variables, such as the lifestyle factors presented above, resulting in a range of solutions. All lifestyle factors were tested for their input/predictor importance. The variables that contributed the most were *Healthy lifestyle, Exercise*, and *Eating.* First, the two-step cluster identify groupings based on a distance measure. This ratio should preferably be below 2 but 3 is acceptable. Present solution had a ratio at 2.5 and was thereby accepted. Secondly, a hierarchical clustering created a range of solutions which then were reduced on the basis of Schwarz’s Bayesian information criterion (BIC) – resulting in 4 clusters. The silhouette measure of cohesion and separation was 0.7, suggesting that the within-cluster and the between-cluster distances are valid.^29^ All analysis was performed with the statistical program SPSS 26.

Worth noticing is that n in for example, Table 3 is based on those who said Yes (changes were made) in regard to a healthier lifestyle, exercise and/or eating more while all who answered Yes or No to the 3 variables are included in the cluster analysis. Since these analysis is based on the variables found to contribute the most the other lifestyle changes is excluded. Therefore, the n is lower than the study sample.

## Results

The respondents’ background is measured at baseline. Since most were middle-aged when quitting smoking the level of education is considered to be probably the same at follow-up.

In the sample most have a high educational level, most were middle-aged when quitting smoking, so now retired, and there are more women than men.

Table 2 shows the frequency of alcohol consumption, as well as binge drinking, at first and second data collection.

As shown, there are more abstainers at follow-up but an increase of individuals who drink several times a week and even daily. Considering binge drinking, there is an overall decrease at follow-up and even though statistically non-significant this gives an indication that drinking habits have changed, especially considering the more hazardous binge drinking.

At baseline, respondents were asked whether they had made any lifestyle changes when quitting smoking. At follow-up, they were asked whether these changes had remained intact in their everyday life.

Table 3 shows that most common when quitting smoking at baseline was, in retrospect, to adopt a healthier lifestyle overall; second, to increase the quota of physical training, followed by eating more. Many had also eased into this new lifestyle on a permanent basis, making it part of their daily life.

The two-step cluster analysis identified 4 profiles and the size and percentage of each cluster as well as the distribution of sex and health behaviors in each is shown in Table 4.

**Table 4.** Health behaviors in each cluster. % of cluster membership.

Cluster	n	%	Sex	Eating	Healthy lifestyle	Exercise
			(W = women, M = men)			
Healthy lifestyle and exercise	160	32.4	W 60.6%	0%	78.8%	45.6%
Women no change	155	31.4	W 100%	0%	0%	0%
Men no change	134	27.1	M 100%	0%	0%	0%
Eating	45	9.1	M 53.3%	100%	28.9%	20.0%

### Healthy lifestyle and exercise

Representing the largest group (n = 160) this first cluster is slightly overrepresented by women. These respondents had not continued to eat more but a majority had adopted a healthier lifestyle and about half had kept physical exercise as part of their regular routines.

### Women without change

The second cluster (n = 155), is dominated by women who had neither continued to eat more food/sweets, nor increased their exercise, nor adopted an overall healthier lifestyle with respect to sleeping habits, diet or leisure time.

### Men without change

The third cluster (n = 134) is dominated by men who, like the women in cluster 2, had not implemented any of the suggested lifestyle factors in their everyday life.

### Eating

The last cluster (n = 45) consists of some more men who had kept their new eating habits, that is, eating more food and sweets. About every fourth also had adopted a healthier lifestyle and every fifth had continued to exercise.

This analysis identified typical groupings of successful quitters. The largest group had implemented a new and healthier lifestyle, however the 2 following groups shows that a long-term stability can be achieved without any changes – and that this is quite common.

## Discussion

This study aimed to examine the association between reported changes in lifestyle and long-term smoking cessation. Among those who made any lifestyle change(s) in connection with smoking cessation, most adopted a healthier lifestyle comprising several different factors, but with the difference of some increasing and others not at all focusing on exercise. Those who did include exercise as a great part of the new healthy lifestyle were mostly women, not unlikely in an effort to counteract any weight gain. Women tend to gain more weight than men when quitting smoking,^23^ and are more likely to resume smoking in order to lose weight again.^30^ So, in order not to relapse, an option would be to increase physical training, which has positive effects on withdrawal symptoms^17,18^ along with increasing the chances for long-term smoking cessation.^22^ Moreover, exercise has a positive influence on mood,^18^ something that facilitates non-relapse for women, while the reverse is true for men, where a positive mood is more likely to lead to relapse.^31^

Quite many also embodied the new healthier lifestyle on a permanent basis. Initially perhaps to dampen withdrawal symptoms, but later on for sustaining overall well-being, with smoking clearly not being consistent with a healthy lifestyle. People who drink alcohol often smoke as well and vice versa, that is, these behaviors tend to go hand in hand. This may also be true for more healthy behaviors; that individuals who adopt a healthy lifestyle in one respect might do so in others as well. There turned out to be more alcohol abstainers at follow-up, and since even low alcohol consumption has been shown to decrease the chance of maintaining smoke cessation over time,^27^ this could be one explanation. On the other hand, there were also more respondents at the time of the second data collection who drank several times a week or daily. We surmise that this is probably due to the high mean age of the sample; older persons in Sweden today tend to drink more than previous generations^32^ and as most were retired at follow-up they might choose to enjoy their golden age in such a way.

Four distinct profiles were identified, whereof the dominating group was in majority when it comes to adopting a healthier lifestyle, including exercise. The 2 following profiles were mostly composed of women respectively men who hadn’t made any permanent life-style changes at all. This is in line with previous research concluding that self-managed smoking cessation is relatively common (see e.g. a population-based study on self-managed smoking cessation^33^ and a study on self-change from addictive behaviors^34^) and to make changes in direct connection with the cessation might be enough.

Eating more, both food and sweets, was a choice made by slightly more men than women. An easy supposition is that cigarette smoking cannot be considered part of a healthy lifestyle, but neither is overeating. Those who gained weight would do well to focus on exercise as a counterbalance, and actually exercise and an overall healthy lifestyle is part of this profile.

### Limitations and strengths of the study

The results should be viewed in the light of some limitations. First, the Monitor data is thought to constitute a representative sample of the Swedish population. However, the research company was merely commissioned to deliver data from 1500 respondents each month and those who could not be reached were simply replaced with others. The missing data increased over time and was about 60% in 2010.^35^ This circumstance raises the question of representability, but as stated in Section 2 no difference between non-respondents and respondents was found when it comes to tobacco use. Second, the smoking cessation and experiences of becoming smoke free might have been dated quite far back in time already when the first survey was carried through. Retrospective data is always afflicted with recall effects. However, information on smoking status^36^ has been found to be quite accurate in retrospective research. Thirdly, most of the respondents were not only long-term smoke free but also long-term nicotine free, meaning that comparative analyses between the nicotine free and the nicotine users are aggravated. Lastly, the non-use stability is more long-term than expected, but it may be that relapses are more prevalent among the dropouts.

In spite of such limitations these data are rather unique in a Swedish context, not least due to the high response rate at baseline and to the follow-up 7 years later.

## Conclusions

In this study we aimed at identifying some key elements of eventual changes in this smoke-free life. Understanding of the eventual impact of such factors is an important step toward providing concrete advice for a continued smoke-free life. First, contrary to expectation, most long-term smoke-free individuals did not report on major changes in their lifestyle. But even if most respondents only made lifestyle changes in direct connection with smoking cessation, an overall healthier lifestyle, especially including physical exercise, seems to contribute to increase the chances for a long-term smoke-free life, as it both alleviates withdrawal symptoms, prevents weight gain, and contributes to a more positive mood and feelings of well-being.

Sweden, being a welfare state with well-developed health care, not only provides medical treatment but also takes seriously the impact of physical activity on health. E.g., it is possible to get a doctor’s prescription for Physical Activity (FaR) to prevent or treat certain ailments.^37^ Secondly, although more research is needed, results from this study lend support to the conclusion that individuals who want to quit smoking should be encouraged to increase their level of activity and, thirdly, that the health care system should be able to provide support for this both initially and over a period of time to promote a lifestyle commensurate with long-term maintenance of smoke-free living.

There are many other lifestyle related aspects of life, which could be of interest for studies of former smokers, than the factors covered in this study. Lastly, more long-term studies should therefore include indicators of lifestyle habits possibly facilitating a continued smoke-free life. The process of becoming smoke-free and the process to remain smoke-free is a struggle for many. Any leads of how to make this easier on the individual smoker should be of great value for the smoking community as well as for the wider society.

## Supplemental Material

Appendix_xyz49725253e72dc – Supplemental material for Lifestyle and Long-Term Smoking Cessation Click here for additional data file. Supplemental material, Appendix_xyz49725253e72dc for Lifestyle and Long-Term Smoking Cessation by Tove Sohlberg and Karin Helmersson Bergmark in Tobacco Use Insights
