# Supplementary material for: Lifestyle and Long-Term Smoking Cessation
Source: Tob Use Insights. 2020 Oct 13;13:1179173X20963062. doi: 10.1177/1179173X20963062 (PMC7576910; doi:10.1177/1179173X20963062)
Supplement: Appendix_xyz49725253e72dc – Supplemental material for Lifestyle and Long-Term Smoking Cessation [file Appendix_xyz49725253e72dc.pdf]

Appendix

Baseline

Lifestyle factors at baseline (total n=1681, missing=2).

To what extent have the following contributed to a successful smoking cessation?

(not at all, to a small extent, to some extent, to a great extent)

I have….

Changed my social networks/friends

Changed my alcohol habits

Started to use nicotine free snus

Changed my habits from those related to smoking

Avoided situations where smoking is common

Devoted myself to physical training

Introduced a healthier lifestyle in respect of e.g. diet, sleep, and leisure time

Begun to eat more food, pastries, candy, desserts

Other…. (please note what)

Successfully smoke- and nicotine free of the 700 respondents in the follow-up: n=605. All of them answered the question on lifestyle (missing=0).

Changes in lifestyle at baseline which has become a permanent lifestyle adjustment (n= 494)

Follow-up

If you think at the time when you quit smoking …. /and the lifestyle changes you made then….

Did any of these lifestyle changes become a permanent part of your lifestyle?

(yes/no)

I now….

Have new social networks/friends

Have other alcohol habits now

Use nicotine free snus

Have other habits than those related to smoking

Avoid situations where smoking is common

Devote myself to physical training

Have a healthier lifestyle in respect of e.g. diet, sleep, and leisure time

Eat more food, pastries, candy, desserts

Other…. (please note what)
